# Therapeutic Fractional Doses of Ionizing Radiation Promote Epithelial-Mesenchymal Transition, Enhanced Invasiveness, and Altered Glycosylation in MCF-7 Breast Cancer Cells

**DOI:** 10.14293/genint.14.1.002

**Published:** 2023-05-04

**Authors:** Raheem AL-Abedi, Seda Tuncay Cagatay, Ammar Mayah, Susan A Brooks, Munira Kadhim

**Affiliations:** 1 Department of Biological and Medical Sciences, Oxford Brookes University, Oxford, OX3 0BP, UK

**Keywords:** Ionizing radiation (IR), epithelial–mesenchymal transition (EMT), glycosylation, invasiveness, breast cancer

## Abstract

The clinical outcome of radiation therapy is restricted due to the acquired radio-resistance of a subpopulation of tumour cells that may cause tumour relapse and distant metastasis. While the effects of ionizing radiation (IR) such as DNA damage and cell stress are well-documented, the potential role of IR in inducing invasive potential in cancer cells has not been broadly studied, therefore we aimed to investigate it in this study. MCF-7 cells irradiated with 0 Gy (control) or 2 Gy X-ray therapeutic doses of IR were assessed for cell viability, percentage of apoptotic cells, and reactive oxygen species (ROS) levels, DNA fragmentation, Matrigel invasion, assessment of epithelial–mesenchymal transition (EMT) markers and *Helix pomatia* agglutinin (HPA) binding at 30 min, 4- or 24-h post-IR. Reduction in cell viability, increase in apoptotic cells, ROS positive cells, and DNA fragmentation were observed, while functional invasiveness and EMT were exacerbated together with altered glycosylation in MCF-7 cells irradiated with 2 Gy X-ray compared to control cells. These findings indicate that despite the detrimental effects of 2 Gy X-ray IR on MCF-7 cells, a subpopulation of cells may have gained increased invasive potential. The exacerbated invasive potential may be attributed to enhanced EMT and altered glycosylation. Moreover, deregulation of transforming growth factor-beta (TGF-β) following IR may be one of the elements responsible for these changes, as it lies in the intersection of these invasion-promoting cell processes.

## Introduction

The vast majority of cancer patients are treated with radiotherapy during the course of their disease either alone or in combination with other modalities including surgery and chemotherapy.^[1]^ Despite being a valuable tool in cancer therapy, because of inherent or acquired radioresistance in some cancer patients, the benefits of clinical radiotherapy are limited.^[2]^ Radiotherapy resistance occurs frequently in breast cancer patients as manifested by tumour recurrence and distant metastasis following radiotherapy treatment.^[3]^

Radiotherapy uses high-energy ionizing radiation (IR) which causes DNA damage in irradiated cells^[4,5]^ resulting in cell cycle arrest, senescence, and apoptosis as well as chromosomal aberrations and genetic mutations.^[6–8]^ IR can also cause production of reactive oxygen species (ROS), which in turn promotes oxidative damage to the membrane of the cells, instigating cell stress and apoptotic cell death.^[5,9]^

Although it is well established that radiation induces cell death, radioresistant subpopulations of tumour cells may cause tumour relapse. Theys *et al.* suggested that IR may exacerbate the invasive potential of tumour cells via the induction of epithelial–mesenchymal transition (EMT),^[10]^ a cellular mechanism that allows epithelial cells to lose their epithelial characteristics, including cell-to-cell contacts, and acquire a migratory mesenchymal phenotype.^[11]^ EMT is a pivotal process in cancer cell migration and invasion,^[12,13]^ which is regulated through modulatory pathways, which involve transforming growth factor-beta (TGF-β), human snail homolog 1/snail homolog 2 (SNAIL/SLUG), twist-related protein 1/twist-related protein 2 (TWIST1/TWIST2) transcription factors, and zinc finger-homeodomain transcription factor family, as well as noncoding RNAs.^[11]^ Accumulating evidence indicates that EMT can promote radiotherapy resistance as radioresistant cancer cells that survive IR have been observed to exhibit an EMT-like changes with an up-regulation of mesenchymal markers and a loss of epithelial markers.^[10,14–16]^

Aberrant glycosylation is another hallmark of cancer that has been linked to invasion and metastasis and can be detected using lectin histochemistry.^[17]^ An increased display of N-acetylgalactosamine (GalNAc), detected by the lectin *Helix pomatia* agglutinin (HPA) binding, has been linked to poor prognosis and an elevated metastatic capacity in a wide variety of cancers, including those of the breast.^[18–21]^ Previously it was suggested that atypical glycans as well as the altered expression of glycosylation related genes may have implications in cells undergoing EMT. Maupin *et al.* showed that O-glycosylation, the sulfation of GAGs, changes to matrix components, mannose receptors, and specific sialylated structures characterize pancreatic cancer EMT.^[22]^ In addition, a growing number of studies have demonstrated that aberrant glycosylation may be associated with treatment failure in patients following radiotherapy.^[23–25]^ For example, another study showed that altered core 1-type O-glycans expression was correlated with advanced tumour stage, metastasis, and poor survival of laryngeal carcinoma patients.^[26]^

TGF-β, an EMT-promoting cytokine, can promote cancer progression by instigating EMT directly through the activation of EMT transcription factors.^[27]^ Interestingly, Freire-de-Lima *et al.* have shown that TGF-β can also induce the up-regulation of a site-specific O-glycosylation in the IIICS (type III connecting segment) domain of human oncofetal fibronectin, which is one of the core components of the extracellular matrix expressed by cancer cells as well as in embryonic tissues.^[28]^

Although the DNA damaging and cell-killing effects of IR are well understood, the role of IR in the induction of EMT and glycosylation have not been broadly studied. Therefore, this study aimed to investigate the effects of a 2 Gy X-ray therapeutic level of IR on markers of EMT, altered GalNAc glycosylation and the invasive potential of MCF-7 breast cancer cells.

## Materials and Methods

### Cell culture

MCF-7 breast cancer cells were kindly provided by Dr. Joestein Dahle (Institute for Cancer Research, Oslo, Norway). The MCF-7 cells were maintained in Dulbecco’s Modified Eagle Medium/Nutrient Mixture F-12 Ham (DMEM/F12) media (D6421, Sigma, St. Louis, MO, USA) supplemented with 10% fetal bovine serum (FBS) (F7524, Sigma, St. Louis, MO, USA), 2 mM L-glutamine (25030149, Gibco, Invitrogen, Paisley, UK), 1% 250 ng/mL insulin (BioXtra, Sigma, 19278, St. Louis, MO, USA), and 1% (v/v) penicillin/streptomycin (P0781, Sigma, St. Louis, MO, USA). Cells were cultured in a humidified incubator in the presence of 5% carbon dioxide (CO_2_) at 37°C.

### Irradiation

MCF-7 cells (70% confluency) were irradiated with therapeutic dose of 2 Gy X-ray utilizing an MXR321 X-ray machine operating at 250 kV constant potential, 14 mA, with a dose rate of 0.53 Gy/min for 3.56 min. Cells were also sham irradiated with 0 Gy X-ray as a control group. All irradiation experiments were conducted at the MRC Oxford Institute for Radiation Oncology, University of Oxford, UK.

### Cell viability assay

Cell viability of irradiated and control cells at 4 h following irradiation was assayed using a Muse™ Count and Viability Cell Dispersal Reagent and Muse™ Cell Analyzer (MCH100107 and 0500-3115, Merck Millipore, Kenilworth, NJ, USA) as previously described in detail.^[29]^

### Reactive oxygen species assay

The population of cells undergoing oxidative stress was measured using a Muse Oxidative Stress kit (MCH100111, Merck Millipore, Kenilworth, NJ, USA). ROS in irradiated and control cells were detected at 30 min following irradiation as previously described in detail.^[30]^ Briefly, cells were harvested in 1X assay buffer and mixed with 190 μl oxidative stress reagent working solution. Following 30 min of incubation at 37°C, Merck Muse cell analyser was used to analyse cells as ROS^-^ (live cells) and ROS^+^ (cells exhibiting ROS).

### Comet assay

A comet assay was carried out with both irradiated and control cells at 4 h following irradiation as explained in detail previously.^[29]^ Briefly, cells were mixed with 200 μl of 0.6% low melting point agarose (LMPA) (BP165-25, Fisher Scientific, Pittsburgh, PA, USA) and put on 1% normal melting point agarose (NMPA) (A9539, Sigma, St Louis, MO, USA) coated microscope slides. The slides were then kept in cold alkaline lysis buffer at 4°C overnight. Next slides were electrophoresed at 4°C, 19 V, and 300 A, for 30 min. Finally, following neutralizing slides with neutralizing buffer, and washing steps, cells on slides were stained with Diamond Nucleic Acid Dye (H1181, Promega, Madison, MA, USA). Analysis of tail intensities of cells was carried out using fluorescent microscopy and Comet Assay IV Image Analysis Software (Perceptive Instruments, Bury St Edmunds, UK).

### Apoptosis

Irradiated and control cells were harvested and centrifuged at 300 × *g* at 4 h following irradiation. After fixing cells in 3:1 methanol: acetic acid, they were stained with Prolong Gold anti-fade reagent with DAPI (Invitrogen, Waltham, Massachusetts, USA, P36931). Analysis of apoptotic cells were carried out under a Zeiss Axioplan 2 upright light/fluorescence microscope (Zeiss, Jena, Germany) and ProgRes C3 camera (Jenoptik, Jena, Germany) under 400× magnification by counting 500 cells/replicate.

### Invasion assay

In order to measure the functional invasiveness of cells, an invasion assay was carried out at 24 h following irradiation as explained in detail previously.^[31]^ In brief, irradiated and control cells were seeded on Matrigel-coated inserts in serum-free media, and incubated at 37°C. At the end of 24 h, the Matrigel and the media were removed and the membranes were washed with phosphate-buffered saline (PBS) solution. Then membranes were fixed in 3:1 methanol:acetic acid and stained with haematoxylin and eosin before being mounted on microscope slides. An Axioplan Light Microscope (Zeiss) with a colour camera (ProgRes), under 200× magnification was used to score total number of invaded cells.

### Immunocytochemistry and lectin cytochemistry

Irradiated and control cells were analysed by immunocytochemistry for EMT markers E-cadherin and vimentin, and by HPA lectin cytochemistry for GalNAc glycosylation at 24 h post-IR, as explained in detail previously.^[31]^ Briefly, cells were fixed with 4% formaldehyde (P1851, Sigma, St. Louis, MO, USA) at 4°C for 15 min and permeabilized with 0.1% v/v Triton X-100 (T9284, Sigma, St. Louis, MO, USA). Following the washing steps, and blockage of endogenous peroxidase using 3% v/v methanol/hydrogen peroxide, the cells were washed three times with 1X Tris buffered saline (TBS). Subsequently, cells were blocked with 3% w/v bovine serum albumin (BSA) for 30 min. For detection of vimentin and E-cadherin, cells were incubated with either 0.5 μg/ml of rabbit monoclonal anti-E-cadherin antibody (ab76319, Abcam, Cambridge, UK) or 2 μg/ml of rabbit polyclonal anti-vimentin antibody (ab137321, Abcam, Cambridge, UK) at 4°C overnight which was followed by washing steps and incubation with 4 μg/ml donkey anti-rabbit IgG H&L horseradish peroxidase (HRP) (ab6802, Abcam, Cambridge, UK) for 1 h. In order to detect altered glycosylation, cells were incubated with 10 μg/ml biotinylated HPA (L6512, Sigma, St. Louis, MO, USA) for 3 min, which is followed by incubation with 5 μg/ml avidin peroxidase (A3151, Sigma, St. Louis, MO, USA) for 30 min. Cells were finally incubated with DAB peroxidase substrate (SK-4100, Vector Laboratories, Burlingame, CA, USA) and counterstained with haematoxylin. Dehydrated and mounted samples were visualised using an Axioplan Light Microscope (Zeiss) with a colour camera (ProgRes), under 400× magnification. Quantification of labelled cells were done by use of Image J software.

### Flow cytometry

Investigation of the EMT marker protein expression of irradiated and control cells was also carried out by flow cytometry, as described in detail previously.^[31]^ Briefly, following permeabilizing cells with 1% Triton X-100 for 10 min, they were incubated with rabbit monoclonal anti-E-cadherin (ab76319, Abcam) or 2 μg/ml rabbit polyclonal anti-vimentin antibody (ab137321, Abcam) for 1 h at room temperature. After the washing steps, cells were incubated with 2 μg/ml AlexaFluor^®^ 488-conjugated polyclonal goat anti-rabbit IgG (ab150077, Abcam, Cambridge, UK) for 30 min at room temperature. Analysis of cell suspensions was carried out using a Cytoflex 5 flow cytometer and CytExpert 2.1 software (Beckman Coulter, Brea, CA, USA). The data were presented as a histogram of vimentin and E-cadherin positive cells.

### Reverse transcription (RT) and quantitative polymerase chain reaction (QPCR)

RT-QPCR was carried out on irradiated and control cells as previously described^[31]^ using the primers listed in Table 1, in order to assess the EMT markers and EMT-associated transcription factors mRNA levels and internal gene control.

**Table 1:** Forward and reverse primer sequences used for qPCR detection.

Target gene	Primer sequence (5′–3′)
**Vimentin**	F: ATGGCTCGTCACCTTCG
	R: AGTTTCGTTGATAACCTGTCC
**E-cadherin**	F: ACGCATTGCCACATACA
	R: CGTTAGCCTCGTTCTCA
**TGFβ-1**	F: TAAAGGGTCTAGGATGCGCG
	R: GACTTTTCCCCAGACCTCGG
**SLUG**	F: AGCAGTTGCACTGTGATGCC
	R: ACACAGCAGCCAGATTCCTC
**SNAIL**	F: AATCGGAAGCCTAACTACAGCG
	R: GTCCCAGATGAGCATTGGCA
**ZEB1**	F: TCCCTGCCAAGAACAATGATCA
	R: AGGTGATGGGGATGGTGTACTA
**Twist**	F: ACAGCCGCAGAGACCTAAAC
	R: GGCCTGTCTCGCTTTCTCTT
**Act-β-1**	F: TGACCCAGATCATGTTTGAGA
	R: TACGGCCAGAGGCGTACAGC

F: Forward; R: Reverse.

### Western blot

Western blots were performed with the cell lysates derived from control cells and irradiated cells, at 24 h post-IR as described in detail previously.^[31]^ Briefly, 20 μg of whole-cell lysates were separated in Mini-PROTEAN^®^ TGX Stain-Free™ Protein Gels (456-8126, Bio-Rad, Hercules, CA, USA) and transferred onto an Amersham™ Hybond™ polyvinylidene difluoride (PVDF) membrane (10600090, GE Healthcare, Little Chalfont, UK). Next, 5% BSA was used to block the membranes, which were then incubated with rabbit polyclonal anti-vimentin (ab137321, Abcam), rabbit monoclonal anti-E-cadherin antibody (ab76319, Abcam) or anti-TGFβ-1 (ab179695, Abcam, Cambridge, UK), at 1:500 dilution, overnight. Following the washing steps, membranes were incubated with goat polyclonal antibody to rabbit IgG-H&L (Alexa Fluor^®^ 488, Abcam) for 1 h at 1:1000 dilution. Visualization was achieved via the use of the Chemi™Doc MP Imaging system (Bio-Rad, Hercules, CA, USA) and analysis was carried out by Image Lab 4.1 software.

### Statistical analysis

For HPA binding and EMT marker assays, the significance of the ratio of the percentage of labelled cells to total cells was subjected to Fisher’s exact test. An invasion assay was also evaluated by Fisher’s exact test. The significance of apoptosis assay data was evaluated by Student’s t-test. For viability, a ROS assay and comet assays statistical significance was determined via the Mann–Whitney U test. Western blot band intensities and RT-QPCR results were represented as mean ± standard deviation (SD). Experiments were carried out in triplicate. Data were accepted statistically significant for *P*-values lower than 0.05 (*P < 0.05, **P < 0.001, ***P < 0.0001).

## Results

In order to investigate the DNA damaging and cell-killing effects of IR on breast cancer cells, MCF-7 cells irradiated with 0 Gy (control) or 2Gy X-ray were assessed for cell viability, percentage of apoptotic cells, ROS^+^ cells, and DNA fragmentation either at 30 min and 4 h post-IR.

**Figure 1:**
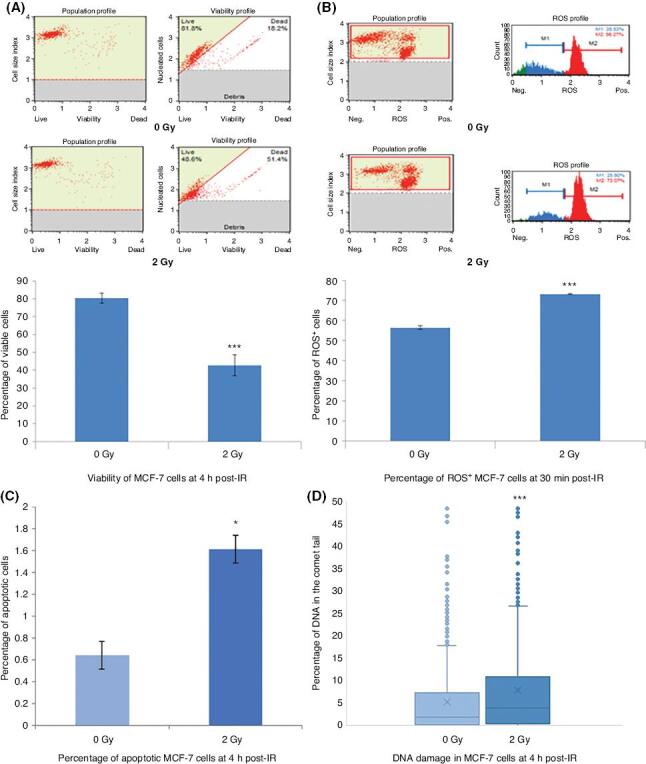
Cell viability, ROS positivity, DNA damage, and apoptosis percentages at 30 min and 4 h following 2 Gy X-ray or sham irradiation of MCF-7 cells. The data are presented as the percentage of (A) cell viability, (B) ROS^+^ cells, (C) apoptotic cells, (D) DNA damage via the comet assay. The error bars represent the SD of cell viability, ROS^+^, DNA damage and apoptosis (*P ≤ 0.05, ***P ≤ 0.0001). The experiments were carried out in triplicate.

A viability assay demonstrated a significant reduction in the percentage of viable cells in 2Gy irradiated MCF-7 cells compared to a sham irradiated control group as shown in Figure 1(A). The percentage of ROS^+^ cells was increased in MCF-7 cells group irradiated with 2 Gy X-ray compared to the control cells as detailed in Figure 1(B). Parallel with these findings, the percentage of apoptotic cells was increased in cells irradiated with 2 Gy X-ray compared to the sham irradiated cells [Figure 1(C)]. Total DNA damage was investigated via the comet assay, where the percentage of DNA in the comet tail indicating DNA damage was increased significantly in the 2 Gy X-ray irradiated cells compared to the control group as depicted in Figure 1(D).

**Figure 2:**
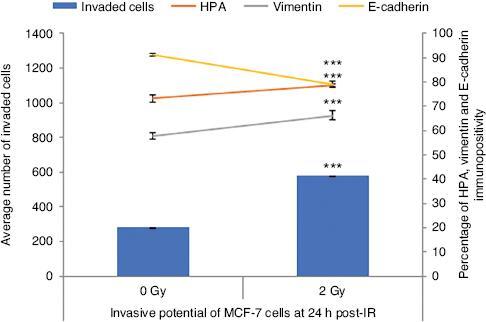
Invasive potential of MCF-7 cells shown by the Matrigel invasion assay, glycosylation, and EMT characteristics following 24 h of irradiation. The data were presented as the mean of a total number of invaded cells, and the percentage of HPA and EMT markers (vimentin and E-cadherin) positive cells. The error bars represent the SD of invasive cells and the percentage of HPA and EMT markers of three independent experiments (***P ≤ 0.0001).

The impact of the 2 Gy X-ray therapeutic level of IR on the invasive potential of MCF-7 cells was investigated using Matrigel invasion and assessment of EMT marker, and HPA binding 24 post-IR, where functional invasiveness, expression of EMT markers, and the glycosylation status of the cells were evaluated, respectively. Data showed a significant increase in the number of invaded MCF-7 cells through the Matrigel transmembrane system after IR exposure as presented in Figure 2. HPA labelling demonstrated a significant increase in the percentage of HPA positive MCF-7 cells when they were irradiated with 2 Gy X-ray compared to sham-irradiated control cells. Moreover, MCF-7 cells irradiated with 2 Gy X-ray showed a significantly increased vimentin immunopositivity and reduced E-cadherin immunopositivity indicating an increase in EMT-like changes.

**Figure 3:**
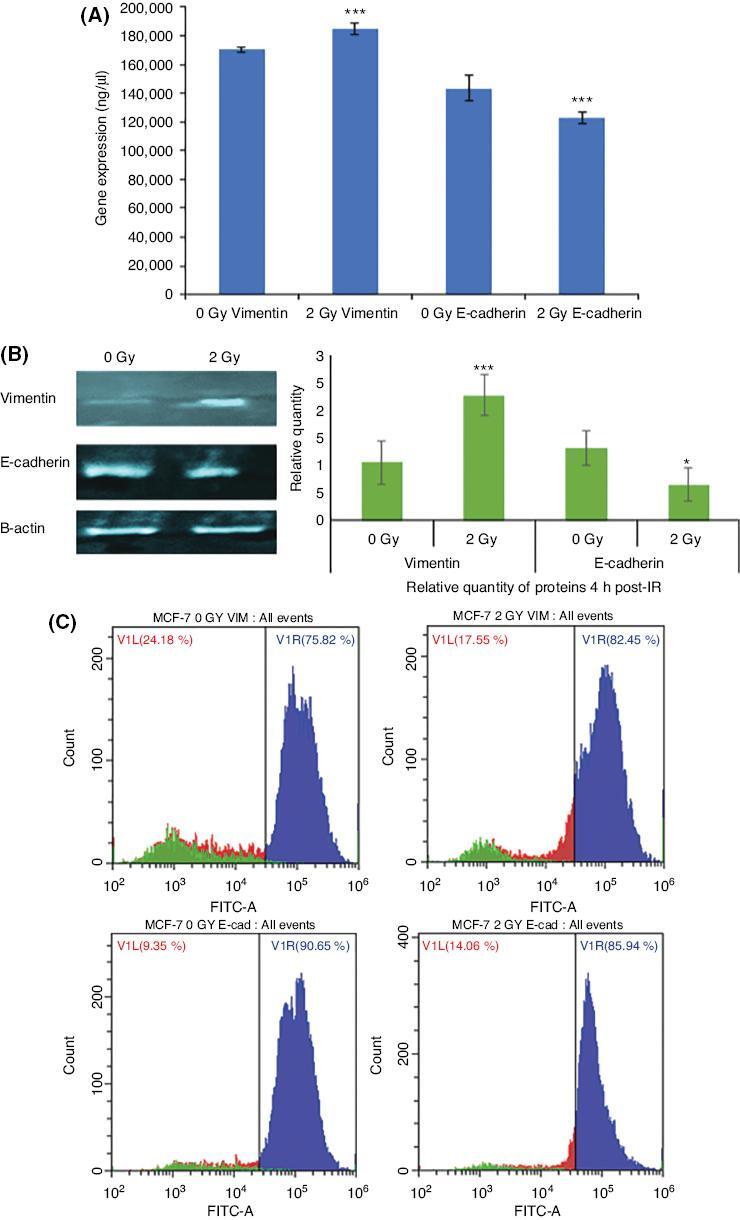
Comparison of vimentin and E-cadherin expression MCF-7 cells at the mRNA and protein levels. (A) qPCR analysis showing vimentin and E-cadherin expression in MCF-7 cells. The error bars represent SD of the mRNA expression. The experiment was carried out in triplicate. (B) Representative Western blot analysis showing E-cadherin (97 kDa) and vimentin (54 kDa) protein levels in MCF-7 cells. (C) Flow cytometry analysis of vimentin and E-cadherin in MCF-7 cells. Data represent three independent experiments (*P ≤ 0.05, ***P ≤ 0.0001).

Next, in order to confirm the augmentation of EMT-like changes in irradiated MCF-7 cells that were observed with EMT marker assay, we investigated expressional changes in EMT markers and transcription factors following 24 h of irradiation. qPCR and Western blot analysis results both showed that there was a significant upregulation of vimentin both at RNA and protein levels, whereas E-cadherin was downregulated in 2Gy X-ray irradiated MCF-7 cells compared to sham irradiated MCF-7 cells [Figure 3(A), (B)]. Similarly, flow cytometry analysis results [Figure 3(C)] showed a significantly increased vimentin immunopositivity and reduced E-cadherin immunopositivity in the 2 Gy X-ray irradiated MCF-7 cells compared to the sham irradiated MCF-7 cells.

**Figure 4:**
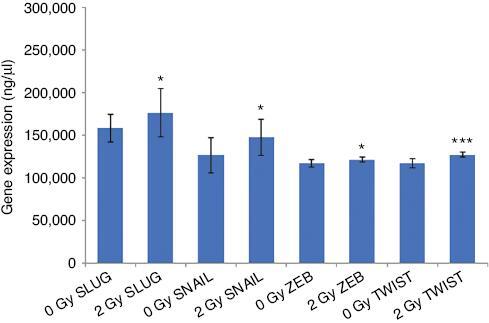
qPCR analysis showing expression of EMT coupled transcription factors SLUG, SNAIL, ZEB, and TWIST in MCF-7 cells irradiated with 0 Gy or 2 Gy X-ray. The error bars represent SD of the mRNA expression (*P ≤ 0.05, ***P ≤ 0.0001). The experiment was carried out in triplicate.

The expression of selected EMT-associated transcription factors was investigated at the mRNA level. qPCR analysis showed that SNAIL, SLUG, (Zinc finger E-box binding homeobox 1) ZEB1, and TWIST were all significantly increased 24 h after 2 Gy X-ray irradiation compared to sham-irradiated MCF-7 cells, as depicted in Figure 4.

**Figure 5:**
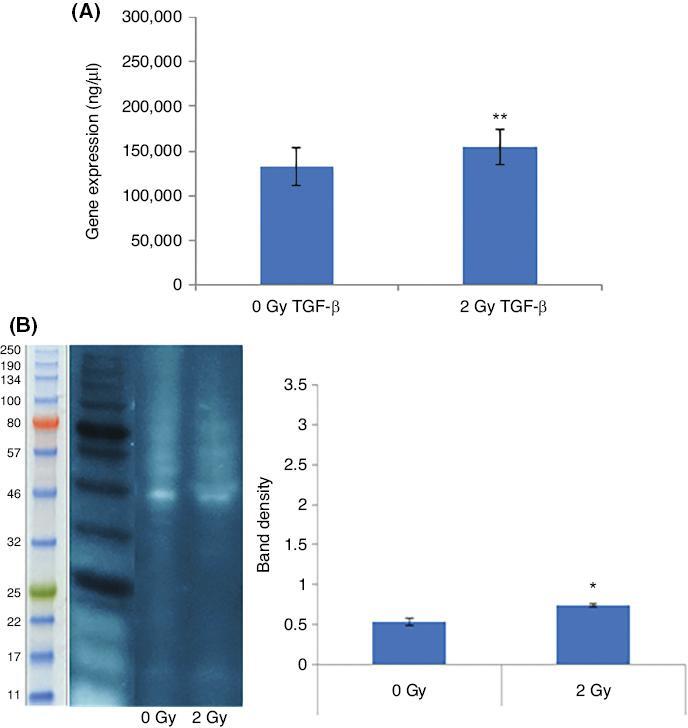
Comparison of TGF-β expression at the mRNA and protein levels in the MCF-7 cells irradiated with 2 Gy X-ray or sham irradiated. (A) qPCR analysis showing TGF-β mRNA expression in MCF-7 cells. The error bars represent SD of the mRNA expression. The experiment was carried out in triplicate. (B) Western blot analysis image shows TGF-β (44 kDa and 12.5 kDa) protein levels in MCF-7 cells. The bar diagram shows the TGF-β protein levels measured as band density. The error bars represent the SD of protein expressions of three independent experiments (*P ≤ 0.05, **P ≤ 0.001).

Finally, observation of a consistent change in EMT markers and EMT promoting transcription factors in MCF-7 cells has driven us to investigate the expression of TGF-β, which is also an important cytokine and reported to influence glycosylation. qPCR analysis and the Western blot analysis [Figure 5(A) and (B), respectively] showed that the TGF-β mRNA and protein expressions were increased in the 2 Gy X-ray irradiated MCF-7 cells compared to the sham-irradiated MCF-7 cells.

## Discussion

Clinical radiotherapy shows limited benefit in some cancer patients due to inherent or acquired radioresistance,^[2]^ while radiotherapy resistance is also common in breast cancer patients as evidenced by tumour relapse and distant metastasis.^[3]^ Therefore, investigation of the mechanisms driving tumour relapse following radiotherapy has critical importance in order to overcome the failure in radiotherapy in breast cancer and improve disease outcomes and increase survival benefit.

In the current study, as expected, we observed that a 2 Gy therapeutic dose of IR had significant DNA damaging and cell-killing effects, as manifested by the results of cell viability, percentage of apoptotic cells, ROS positivity, and the comet assay in MCF-7 cells at 30 min and 4 hpost-irradiation (Figure 1). Despite the cell-killing effects of IR, previous data suggest that the invasive potential of tumour cells can be enhanced by IR exposure.^[10,32]^ Therefore, we also aimed to investigate the invasive potential of MCF-7 cells following 2 Gy X-ray irradiation. Interestingly, our findings indicated that cancer cells that survived X-ray treatment demonstrated a significant change in their invasive potential, as depicted in Figure 2. Cells were significantly more invasive following 2 Gy X-ray irradiation compared to the sham irradiated counterparts.

Aberrant glycosylation, demonstrated by binding of the lectin HPA, is linked to invasion and poor prognosis in many human cancers, including breast cancer.^[21]^ Importantly, a growing number of studies have demonstrated that aberrant glycosylation may be associated with treatment failure in patients following radiotherapy.^[23,24]^ As shown in Figure 2, we observed that cells exposed to IR have increased HPA immunopositivity. Besides, we observed a shift to an EMT-like profile as the cells showed a reduction in E-cadherin but an increase in vimentin immunopositivity, shown by the EMT marker assay (Figure 2) and also Western blot analysis and flow cytometry [Figure 3(B) and (C), respectively]. The same trend was confirmed with qPCR showing the change in expression of these two markers is at the mRNA level [Figure 3(A)].

Our findings regarding reduction in E-cadherin are not only important because it is a key EMT marker, but also because of its central importance in radiation response. Theys *et al.* showed that sparsely seeded MCF-7 are more radioresistant compared to dense MCF-7 cultures following IR treatment and that mesenchymal MDA-MB 231 cells could be radio-sensitized by the reconstitution of E-cadherin expression.^[10]^

The loss of EMT epithelial gene expression and activation of a mesenchymal phenotype involves the core set of EMT-coupled transcription factors, SNAIL, SLUG, TWIST, and ZEB1.^[33]^ SNAIL and SLUG are known as strong repressors of E-cadherin expression.^[34]^ TWIST has been shown to bind to the E-cadherin promoter, regulating E-cadherin promoter activity and its expression shown to have inverse correlation with E-cadherin expression in clinical breast cancer samples.^[35]^ ZEB1 represses EB1 transcription of E-cadherin through E-box sequences in its promoter region.^[36]^ In the study reported here, as shown in Figure 4, SNAIL, SLUG, TWIST, and ZEB1 are all upregulated at 24 h following 2 Gy X-ray irradiation of MCF-7 cells, which might have contributed to the downregulation of E-cadherin in those cells compared to sham-irradiated cells.

EMT-promoting cytokine TGF-β expression was enhanced at mRNA [Figure 5(A)] and protein [Figure 5(B)] levels following irradiation with 2 Gy X-ray in MCF-7 cells. TGF-β plays a prevalent role in EMT regulation^[27]^ and the EMT-like changes observed in MCF-7 cells following X-ray irradiation in this study may be partially attributed to this upregulation. Besides, the EMT programme has been linked to increased resistance to ionizing radiation via complex molecular mechanisms. IR activates the TGF-β pathway through tyrosine kinase activation of TGF-β receptor I and II, which in turn phosphorylates Smad2/3 promoting EMT and radioresistance.^[37,38]^

Overall, in this study, we have shown that although the 2 Gy X-ray therapeutic level of IR has detrimental effects on MCF-7 cells including DNA damage and apoptosis, there may be a subpopulation of cells that have gained increased invasive potential. The exacerbated invasive potential may be the collective result of enhanced EMT and altered glycosylation. Moreover, deregulation of TGF-β following IR may be one of the elements responsible for these changes, as it lies in the intersection of these invasion-promoting cell processes. These findings may have important implications in terms of understanding radiation therapy resistance and developing new treatment strategies in breast cancer.
